# Filling the mechanistic and methodological gaps for predicting cytokine release syndrome in humans

**DOI:** 10.3389/fphar.2026.1839092

**Published:** 2026-06-16

**Authors:** Laure-Alix Clerbaux, Miriam Alb, Birgit Fogal, Thomas Hartung, Virginia K. Hench, Hannah Morgan, Felicity N.E. Gavins, Birgitte Lindeman, Luigi Margiotta-Casaluci, Marek Ostaszewski, Kristin Reiche, Magdalini Sachana, Clemens Wittwehr, Katherina Sewald

**Affiliations:** 1 Institute of Experimental and Clinical Research, UCLouvain, Brussels, Belgium; 2 Universitätsklinikum Würzburg, Med. Klinik und Poliklinik II, Lehrstuhl für Zelluläre Immuntherapie, Würzburg, Germany; 3 Sanofi, Cambridge, MA, United States; 4 Johns Hopkins University, Baltimore, MD, United States; 5 University of Konstanz, Konstanz, Germany; 6 Open BioData Modeling, Raleigh, NC, United States; 7 Novartis, Basel, Switzerland; 8 Centre for Inflammation Research and Translational Medicine (CIRTM), Department of Biosciences, Brunel University of London, Greater London, United Kingdom; 9 Norwegian Institute of Public Health, Division of Climate and Environmental Health, Oslo, Norway; 10 Institute of Pharmaceutical Science, Faculty of Life Sciences and Medicine, King’s College London, London, United Kingdom; 11 Luxembourg Centre for Systems Biomedicine, University of Luxembourg, Esch-sur-Alzette, Luxembourg; 12 Fraunhofer Institute for Cell Therapy and Immunology IZI, Leipzig, Germany; 13 Environment Health and Safety Division, Environment Directorate, Organisation for Economic Co-operation and Development (OECD), Paris, France; 14 European Commission, Joint Research Centre (JRC), Ispra, Italy; 15 Fraunhofer Institute for Toxicology and Experimental Medicine, Hannover, Germany

**Keywords:** adverse outcome pathway, cytokine release syndrome, immune-related adverse event, new approach methodologies, regulatory

## Abstract

Cytokine release syndrome (CRS) is a severe immune-mediated toxicity frequently associated with immunotherapies such as CAR-T cells and bispecific T-cell engagers. Predicting CRS in humans remains difficult because current animal models and *in vitro* systems do not adequately capture the complex, systemic, and patient-specific nature of pathological inflammation. This perspective highlights key mechanistic and methodological limitations in current immunotoxicology approaches and proposes an immune-related Adverse Outcome Pathway (irAOP) framework adapted for biotherapeutics. The irAOP approach structures CRS as a sequence of interconnected key events (KEs), including immune cell activation, recruitment of pro-inflammatory cells, and excessive cytokine release. These KEs are harmonized from existing AOPs to distinguish pathological inflammation from normal immune responses. Emphasis is placed on downstream effects such as endothelial activation and vascular leakage, which are central to CRS severity. Clinical manifestations - including fever, hypotension, hypoxia, edema, and multi-organ dysfunction - are linked to late-stage KEs, enabling a mechanistic bridge between molecular events and patient outcomes. Insights from COVID-19-related irAOP development and international initiatives like the imSAVAR consortium inform a roadmap for improving CRS prediction. This includes defining context of use, strengthening mechanistic evidence through quantitative key event relationships, and advancing human-relevant New Approach Methodologies (NAMs). By integrating advanced *in vitro* and *ex vivo* systems within an irAOP framework, fragmented data can be transformed into coherent, decision-relevant tools. Overall, this strategy supports a shift toward predictive, human-centered approaches for assessing and managing CRS risk in immunotherapy.

## 1 Introduction

### 1.1 Regulatory challenges in prediction of immune-related adverse events induced by cellular immunotherapies

Immune-modulating therapies are central to modern medicine but often induce immune-related adverse events (irAE), a term widely used in the immunotherapy field to describe unintended and potentially severe immune-mediated toxicities (Naidoo et al., 2023). One prominent example of such an irAE is cytokine release syndrome (CRS) (Postow et al., 2018; Neelapu et al., 2018; Choi and Lee, 2020; Morris et al., 2022). CRS is an acute systemic inflammatory reaction caused by excessive cytokine production. Clinically, it manifests with high fever, hypotension, hypoxia, and, in severe cases, multi-organ dysfunction (Shimabukuro-Vornhagen et al., 2018).

Conventional animal models fail to predict this outcome reliably, largely due to fundamental species-specific differences in immune system architecture and function (Mestas and Hughes, 2004), underscoring the critical need for human-based assays. This limitation is particularly pronounced in the chimeric antigen receptor (CAR)-T cell field, where preclinical studies are predominantly conducted in severely immunocompromised NOD. Cg-Prkdcscid Il2rgtm1Wjl/SzJ (NSG®) mice. In these models, CAR-T cells are often the only functional immune effector population present unless additional immune cells (e.g., human peripheral blood mononuclear cells (PBMCs) are engrafted. Consequently, key downstream immune interactions required for the manifestation of CRS cannot be adequately captured, and later key events (KEs) of the CRS-related immune adverse outcome pathway (irAOP), such as systemic cytokine amplification, vascular dysfunction, and multi-organ involvement, cannot be meaningfully studied.

While the European Medicines Agency (EMA) does not formally endorse a specific PBMC-based cytokine release assay, its post-TGN1412 guidance for first-in-human studies emphasizes MABEL-based dose selection and *in vitro* cytokine release testing to assess the risk of cytokine release syndrome (Vessillier et al., 2015). These assays measure cytokine secretion by human PBMCs in response to a test compound, providing an indication of its potential to activate T cells and trigger cytokine release. However, such PBMC-based assays do not capture individual patient-specific factors, tumor-related features such as tumor mass, or the full clinical manifestation of CRS described above. In addition, cytokine levels measured in these assays are often poorly correlated with clinical CRS severity and are therefore of limited value for identifying patients at highest risk. This limitation is further challenged by the strong dependence of assay outcomes on experimental design, including variables such as cellular composition, target burden, effector-to-target ratios, stimulation conditions, assay timing, and endpoint selection. Together, these factors reveal both mechanistic and methodological gaps in predicting severe outcomes such as high fever, hypotension, hypoxia, and multi-organ dysfunction, and indicate that other patient- and disease-related variables may be more informative predictors of clinical risk.

To better predict CRS in patients, clinical symptoms need to be mapped back to their underlying mechanisms. Aligning symptomatology with perturbation of key biological effects such as cytokine-mediated endothelial cell activation, vascular leakage, and hypothalamic fever is essential for improving the anticipation of CRS risk related to immunomodulatory biotherapeutics. Establishing this mechanistic picture will also enable the alignment of relevant, and potentially personalized, human-based methodologies capable of capturing the sequential events leading to CRS.

Importantly, CRS is not the only clinically relevant immune-related toxicity associated with cellular immunotherapies. A particular challenge of CAR-T cell therapies, and in some cases also bispecific T-cell engagers, is immune effector cell-associated neurotoxicity syndrome (ICANS), an acute neurotoxic syndrome that may occur in the setting of immune activation. ICANS has been associated with systemic inflammatory responses, endothelial cell activation, blood-brain barrier dysfunction, and neuroinflammation (Gust et al., 2017; Norelli et al., 2018). Its clinical manifestation is highly variable between patients, indicating that additional disease-, treatment-, and patient-specific factors are likely to shape its development and severity.

As with CRS, the lack of experimental models that reliably predict individual human responses has substantially constrained mechanistic understanding and risk anticipation for ICANS. Current preclinical models do not adequately capture the complex neuroimmune and neurovascular interactions thought to underlie this toxicity. Emerging human-based approaches, including patient-derived cytokine release assays combined with advanced *in vitro* brain models such as organoids or blood-brain barrier-relevant systems, may provide a promising basis to extend the irAOP framework toward neurotoxicity-related adverse outcomes and to support more predictive safety assessment of CAR-T cell therapies.

### 1.2 A mechanistic framework and human-relevant NAMs for predicting CRS

Adverse Outcome Pathways (AOPs) structure knowledge by linking molecular initiating events (MIEs) through key events (KEs) to adverse outcomes (AOs) (Ankley et al., 2010; Villeneuve et al., 2014). While this concept originated in the context of industrial chemicals, its value in drug development is increasingly recognized. As inflammation underpins many AOs, inflammation-related KEs have been proposed as cross-cutting “hub” KEs (Villeneuve et al., 2014). To date, immune-related AOPs (irAOP) have been more extensively developed for chemical stressors than biological interventions. Although inflammatory responses differ markedly depending on the initiating stressor, affected tissue, exposure route, kinetics, immune-cell composition and host context, several core inflammatory processes are shared across many adverse outcome pathways. These include activation of immune cells, recruitment of pro-inflammatory immune cells and increased production of pro-inflammatory mediators. In the CRS context, these upstream inflammatory hub KEs are mechanistically connected to downstream events such as endothelial activation and vascular dysfunction, which are central to the clinical severity of CRS. In this sense, broadly defined inflammatory hub KEs provide a reusable scaffold for irAOP development. However, reuse of these KEs does not imply that chemical, infectious and biotherapeutic stressors follow identical pathways or produce equivalent outcomes. For CRS induced by T-cell engagers or cellular immunotherapies, the relevant context of use is defined by modality-specific MIEs, exposure scenarios, immune-cell interactions, systemic amplification dynamics and patient-specific modulating factors. These elements determine whether shared inflammatory hub KEs remain part of a controlled immune response or progress toward pathological systemic inflammation. During the COVID-19 pandemic, irAOPs were developed to capture the complex immune response triggered by SARS-CoV-2 (Nymark et al., 2021). This international effort has led to the proposal of an irAOP leading to CRS as a dedicated outcome category to differentiate excessive, damaging inflammation from normal host defense (Nymark et al., 2024). More recently, the imSAVAR consortium aimed at extending irAOP to biotherapeutics as this represents a major strategic opportunity to advance predictive immunotoxicology (Alb et al., 2024; Roser et al., 2024; Clerbaux et al., 2024). Regulatory acceptance of human-relevant New Approach Methodologies (NAMs) at EMA requires that each method be evaluated within a clearly defined context of use (here immunotherapies). Different levels of evidence and validation will be expected depending on whether a method supports pivotal safety decisions or informs efficacy. Acceptance criteria therefore differ, and the regulatory expectations for NAMs vary accordingly. Here, we would like to capture the pivotal safety decisions for CAR-T cells inducing CRS.

While the recently published explorable CRS AOP model by Mazein et al. provides an important computationally accessible representation of mechanistic knowledge for CRS induced by immunomodulatory biotherapeutics and cellular therapies, the present perspective addresses a complementary question: how such an irAOP can be operationalized for translational safety assessment. We therefore synthesize coordinated contributions from international experts spanning regulatory science, toxicology, NAM development, computational biology, and clinical immunology to examine how CRS-irAOPs and appropriate human-relevant NAMs can be leveraged as translational frameworks for biotherapeutic stressors. The focus is on the methodological and regulatory gaps that currently limit their use in drug development, including fit-for-purpose context definition, mapping of NAMs to MIEs and KEs, development of quantitative or semi-quantitative KERs, consideration of clinically relevant modulating factors, and integration of NAM outputs into IATA/NGRA strategies. Thus, the aim is not to duplicate existing CRS AOP models, but to distill expert insights into a unified scientific roadmap for their practical application to T-cell engagers and cellular immunotherapies.

## 2 Proposed irAOP leading to CRS induced by cellular immunotherapies

### 2.1 Molecular initiating events (MIE) for cellular immunotherapies

As discussed in Clerbaux et al. (2024), the common challenges of incorporating non-chemical stressors within the AOPs appear mainly related to the initial interactions of the stressor with the biological system. Adaptations need to be implemented to accommodate those considerations for MIE depending on the stressor.

Biotherapeutics, such as CAR-T cells or monoclonal antibodies, which directly activate immune cells, pose challenges that differ fundamentally from those associated with inert chemicals. Therapeutic efficacy is often intrinsically linked to immune activation (e.g., cytotoxic T cell responses, cytokine production). Hence, irAOP must help distinguish beneficial from harmful inflammation and support the definition of acceptable windows of immune activation rather than simply “on/off” toxicity. Consequently, their MIEs and initial KEs must be adapted to reflect their biological specificity. CAR-T cells, for instance, “do not respect” the classical progression from molecular to cellular to tissue-level events in a simple linear way, but rapidly span multiple levels of organization. CAR-T cells act as self-amplifying biotherapeutics that expand *in vivo*, secrete cytokines and interact with multiple cell types simultaneously. After binding to the antigen expressing cell, CAR-T cells are activated (KEx: activation of immune cells) and then release pro-inflammatory mediators, which then recruit other immune cells that upon activation release pro-inflammatory mediators. Similarly, T-cell engager (TCE) result in activation and proliferation of endogenous T cells, initiating a similar cascade of events. Therefore, the first KE is not a reaction of the biological system the stressor is interacting with, it is a reaction of the cellular stressor itself. We decided not to duplicate KEs with regard to guiding the development of test systems.

### 2.2 Hub inflammatory KEs as central drivers of CRS

**FIGURE 1:**
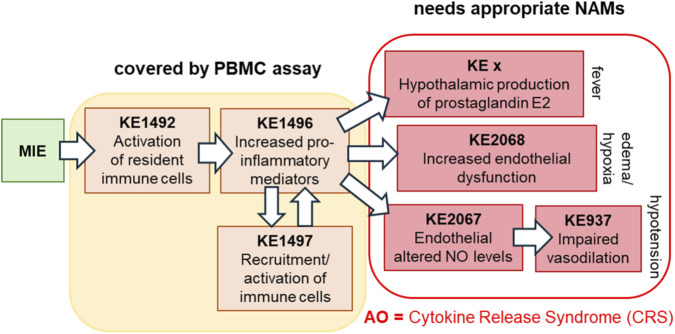
Proposed AOP for CRS. Following the molecular initiating event (MIE), activation of resident immune cells induces pro-inflammatory mediator release and recruitment of additional immune cells, leading to inflammatory amplification. Downstream effects include hypothalamic prostaglandin E2 production and endothelial dysfunction, culminating in CRS.

The hub KEs proposed in Villeneuve et al. (2018a) “*Activation, Resident Immune Cells*” (KE1492), *“Increase, Recruitment of Pro-inflammatory Immune Cell*s” (KE1497), and *“Increase, Pro-inflammatory Mediators*” (KE1496) represent core inflammatory processes that are fundamental to immune activation, independent of the specific tissue or anatomical context in which they occur. These KEs capture generic biological responses that are shared across organs and disease settings and therefore constitute central nodes in inflammatory AOPs, including those leading to CRS (Figure 1).

A critical challenge in applying these KEs to irAOP lies in defining an appropriate level of biological granularity. In the immune system, activation, amplification, and propagation of inflammatory signals are inherently dynamic, context-dependent, and highly individualized. Systemic inflammation, in particular, does not represent a single uniform biological state but rather a spectrum of interacting cellular and molecular processes that vary substantially between individuals (Medzhitov, 2008; Lee and Cron, 2023).

In the proposed harmonised AOP, these KEs are therefore intentionally defined at a relatively broad level. No single experimental readout captures all biologically relevant and complex aspects of immune cell activation, recruitment, or mediator release. Conversely, excessive subdivision of these KEs into multiple narrowly defined measurements risks obscuring their causal role and fails to provide a clear threshold at which cumulative immune perturbations translate into an adverse outcome. Maintaining a higher-level KE definition thus reflects both the current limits of mechanistic resolution and the need for biological coherence when describing systemic immune dysregulation leading to CRS.

While the hub KEs are defined in a tissue-agnostic manner, the distinction between local and systemic manifestations becomes relevant at the level of exposure context and stressor distribution. When immune activation is initiated locally, such as at barrier tissues or within a confined microenvironment, these KEs may initially unfold in a spatially restricted manner, with amplification and propagation determining whether the response remains contained or transitions into systemic inflammation. In contrast, when immune-modulating stressors are introduced directly into the systemic circulation, as is the case for many systemically administered cellular immunotherapies and T-cell engagers, immune activation may emerge system-wide without a clearly separable localized initiation phase. In contrast, locally or intratumorally administered T-cell engagers may initially induce immune activation within a spatially restricted tumor microenvironment. In these cases, progression toward CRS depends on whether local cytokine production and immune amplification propagate into the systemic circulation, which will be influenced by dose, target burden, vascular access, tissue retention and patient-specific immune responsiveness. Thus, local and systemic inflammation should be understood as different exposure- and distribution-dependent modes of KE expression within a shared causal framework, rather than as universally sequential steps.

### 2.3 Systemic cytokine release as a central immune amplification step in CRS

As said, CRS is a *systemic* inflammatory reaction driven by *excessive* cytokine production, and this *systemic* nature is essential to capture. Typically, a progression from local to systemic inflammation precedes the full spectrum of CRS symptoms. For example, in SARS-CoV-2 infection, inflammation originates in the lungs and becomes clinically problematic once it extends into the systemic circulation. This transition from local to systemic inflammation may occur almost instantaneously - meaning may not be relevant to capture - when the stressor is introduced directly into the systemic circulation, as is the case for many immunomodulatory therapies (discussed in the next paragraph).

Moreover, the *excessive* or stormy aspect of the response needs also to be captured. A bidirectional feedback loop between the recruitment of immune cells and the release of pro-inflammatory cytokines reinforces and amplifies the reaction, driving the rapid intensification characteristic of CRS. Hence, this stage in the progression of the syndrome is proposed to be captured by *“Increase, Pro-inflammatory Mediators”* (KE1496) and *“Increase, Recruitment of Pro-inflammatory Immune Cells”* (KE1497), which together form a mutually reinforcing feedback loop amplifying the systemic inflammatory response (Figure 1).

### 2.4 Defining CRS as a set of adverse outcomes (AO)

Clinically, CRS manifests with high fever, hypotension, hypoxia, edema and, in severe cases, multi-organ dysfunction (Shimabukuro-Vornhagen et al., 2018). Defining this constellation of symptoms as a single, uniform AO is not appropriate, nor adequate for predicting using relevant human-based methodologies. Instead, we propose to capture CRS through a set of late KEs that must all be met to predict the clinical phenotype of CRS. In this framework, CRS as an AO is established when the stressor can be shown to trigger key mechanistic events such as fever, vascular leakage, systemic hypotension, and hypoxia (Table 1), collectively capturing the clinical syndrome. Organ-specific vascular leakage leads to inadequate organ perfusion and ultimately to multi-organ dysfunction. In other words, multi-organ dysfunction in CRS primarily results from cytokine-driven endothelial cell activation causing widespread vascular leakage and organ hypoperfusion, rather than from direct cytokine-mediated injury to organ parenchyma. Hence, if vascular leakage induced by a stressor can be measured *in vitro* in an organ-specific manner, the resulting data could reasonably be predictive of multi-organ dysfunction.

**TABLE 1:** Key clinical features of cytokine release syndrome and their underlying organ-specific mechanisms.

Clinical symptoms	Proposed late KE/biological process	Organ(s) affected	NAM readouts	Translational limitation
Fever	Cytokine-induced PGE_2_ production/hypothalamic fever axis	CNS/hypothalamus	PGE_2_ release; COX2 expression in cytokine-stimulated monocytes/macro-phages or transfer assays	Indirect fever modelling; no established AOP-Wiki KE for hypothalamic fever induction
Hypotension	Vasodilation and vascular tone dysregulation	Arterioles/vascular system	NO/nitrite levels; eNOS/iNOS expression; vessel diameter or flow resistance in vascular chips	NO is transient and context-dependent; systemic vasodilation difficult to model *in vitro*
Hypoxia	Endothelial dysfunction and vascular leakage	Lung microvasculature	TEER; dextran leakage; VE-cadherin disruption; ICAM-1/VCAM-1 expression in lung-relevant endothelial systems	Requires lung-specific endothelial models and clinically anchored permeability thresholds
Edema	Cytokine-driven barrier disruption and vascular leakage	Lung, gut, liver and other vascular beds	Barrier permeability; endothelial junction disruption; inflammatory adhesion markers in organ-specific endothelial models	Organ-specific vascular beds differ substantially; systemic fluid shifts are not fully captured

Regarding fever, no KE describing hypothalamic PGE_2_-mediated fever induction is currently available in the AOP-Wiki; such a KE would need to be created. Cytokine release activates PLA_2_ to increase arachidonic acid availability and induces COX-2 expression to enhance its conversion into prostaglandin precursors, collectively driving elevated PGE_2_ synthesis responsible for fever. For vascular leakage, the KE *“Increase, Endothelial Dysfunction”* (KE 2068) is present within an OECD-endorsed AOP (from the irradiation field). This KE includes methodologies to assess endothelial senescence, cell death, and barrier permeability. For vasodilation, relevant KEs exist, namely, *“Altered, Nitric Oxide Levels”* (KE 2067), which is also present within an OECD-endorsed AOP and *“Impaired, Vasodilation”* (KE937), which is under (non-active) development.

## 3 Remaining challenges in developing an irAOP for CRS

### 3.1 Tackling tissue specificity within broad KEs

The need to define tissue-/organ-specific KEs has already been recognized, notably in the context of COVID-19, where the relevance of immune responses was shown to depend on the affected tissue (Nymark et al., 2024). In parallel, improving the completeness of AOP entries in the AOP-Wiki has been identified as a prerequisite for facilitating AOP integration and network building. Likewise, the importance of fully implementing the FAIR (findable, accessible, interoperable, reusable) principles has been emphasized to enhance data accessibility, interoperability, and reusability (Wittwehr et al., 2024; Mortensen et al., 2025). However, the key AOP principle of *reusability* introduces a fundamental tension: while KE reuse favors broadly defined events, AOPs developed for specific contexts of use or fit-for-purpose applications often require KEs to be precisely defined with respect to species, tissue, or organ. Here, we focus on the challenges that are specific to AOPs describing excessive immune responses leading to CRS in humans induced by immunotherapies, such as CAR-T cells and TCEs, where tissue-specific mechanisms are critical for both biological relevance and regulatory applicability.

### 3.2 KERs and quantitative relationships–the key but missing pieces

The need to coordinate and harmonize acceptance of NAMs across regulatory systems was explicitly acknowledged by EMA. However, chemical regulation is guided by the *precautionary principle*, aiming to minimize exposure to any potential hazard. In contrast, in the biomedical field, drug safety assessment is intrinsically based on a *benefit-risk evaluation*, acknowledging that some degree of hazard may be acceptable if therapeutic benefit is significant. This fundamental difference is key to capture when moving towards using AOPs for drugs. Quantitative information is often lacking in AOPs. For regulatory decision-making (dose-response, margin of safety), AOPs need response-response relationships (i.e., quantitative links between KEs). Yet many published AOPs remain qualitative. For a CRS-irAOP, it would be critical to know, for a given biologic dose and exposure scenario, whether upstream immune activation will progress to vascular leakage, systemic inflammation, and CRS. Quantitative implementation of the CRS-irAOP requires the establishment of response-response relationships between upstream and downstream KEs. For example, cytokine concentrations or cytokine patterns generated after immune-cell activation need to be linked to endothelial activation, vascular leakage, fever-related PGE_2_ production, nitric oxide-mediated vasodilation, and thromboinflammatory responses. Such relationships should capture temporal dynamics, thresholds, and tipping points that determine whether an initially intended immune activation progresses toward clinically relevant CRS.

Building on these response–response relationships, the temporal and quantitative dynamics of immune perturbation need to be described within the KERs of the CRS-irAOP. This quantitative aspect is key for capturing pivotal safety decisions but is still an important missing piece in this AOP. We especially need to put effort into the *in vitro* to *in vivo* extrapolation (IVIVE) aspect. An important advantage of biotherapeutics is that their dose and exposure can be precisely controlled, in contrast to environmental or occupational chemical exposures. The route of administration differs also fundamentally between chemicals and biotherapeutics. For chemicals, exposure levels are often unknown and typically occur via oral, dermal, or inhalation routes, leading to an initial local impact (hence local inflammation) that can then spread towards systemic ones. In contrast, biotherapeutics are administered at controlled doses, most often by intra venous injection, which directly shapes their systemic availability. Consequently, systemic inflammation and CRS do not necessarily follow a local-to-systemic progression for many biotherapeutics; rather, acute systemic responses may arise immediately upon administration. Without quantitative or at least semi-quantitative KERs, regulatory applicability is limited. At the same time, this represents a major opportunity for the development of quantitative systems toxicology (QST) models, which could help link dose, exposure, immune activation dynamics, and clinical outcomes in a more predictive manner.

Unlike chemical regulation, the medicinal product lifecycle offers a unique opportunity to refine and calibrate irAOPs using post-marketing pharmacovigilance data, creating a continuous learning cycle between preclinical NAMs, mechanistic AOPs, and real-world clinical outcomes.

### 3.3 Modulating factors participating in the heterogeneity of the outcome

Whatever the stressor, the immune response will also vary based on a number of factors, including existing comorbidities, prior immunomodulating therapies, genetics, sex, age or co-exposure to chemicals. The COVID pandemic clearly highlighted that adverse outcomes are markedly heterogeneous, from asymptomatic to death. Addressing how diverse factors drive excessive inflammatory status for some patients is crucial. Immune responses are modulated by host factors (e.g., genetics, age, comorbidities, immunological history), which are difficult to integrate into a “one-size-fits-all” AOP. Efforts have started to integrate factors modulating the immune response in AOPs (Clerbaux et al., 2022) that will be key to better take into consideration more vulnerable populations. The COVID pandemic showed that different factors modulate individual immune responses (reviewed in Clerbaux et al., 2022). Among those, intrinsic factors such as age and sex strongly modulate how effectively cytokine release can recruit immune cells, with older age and male sex being associated with exaggerated inflammatory responses and higher immune-cell recruitment, thereby increasing risk of severe disease (Klein and Flanagan, 2016; Vemuri et al., 2019; Flanagan and Klein, 2026). Among co-morbidities, obesity alters the inflammatory background, for example, via metabolic and lipid-mediated modulation of immune cell activation, which may amplify cytokine production and enhance immune cell recruitment (Stefan et al., 2021). Pre-existing cardiovascular disease may further exacerbate these effects, as baseline endothelial dysfunction, impaired tissue perfusion, and vascular stress can synergize with cytokine-driven inflammatory signalling, thereby promoting disproportionate immune cell recruitment and worsening inflammatory outcomes (Evans et al., 2020). Gut dysbiosis is more and more acknowledged as modulating systemic inflammation: dysregulated intestinal microbiota can prime immune cells or alter systemic cytokine levels, thereby increasing the recruitment of pro-inflammatory cells in response to the initial cytokine release (Clerbaux et al., 2022). Finally, diet strongly influences basal inflammatory status, or threshold for activation, thereby modulating individual vulnerability to an over-exuberant immune response. Vitamin D deficiency, common in patients with chronic illnesses, including cancer, is particularly prevalent among individuals with reduced sunlight exposure or dietary insufficiency. The active form of vitamin D acts as a potent immunomodulatory hormone, influencing both innate and adaptive immunity. Deficiency can shift the immune system toward excessive inflammation, creating conditions that may exacerbate cytokine-driven responses. The inflammatory prostaglandin PGE_2_ is synthesized from arachidonic acid, an omega-6 fatty acid. In contrast, EPA (an omega-3 fatty acid) gives rise to alternative prostaglandins such as PGE_3_, which are significantly less pro-inflammatory. Thus, the dietary balance of omega-6 and omega-3 fatty acids influences whether cells predominantly generate strongly pro-inflammatory (e.g., PGE_2_) or weaker inflammatory (e.g., PGE_3_) prostaglandins (Calder, 2013; Gutiérrez et al., 2019; Bodur et al., 2025).

In the context of CAR-T cell therapies, however, the influence of those different factors appears more complex and less directly linked to adverse events. While age has a well-documented impact on CAR-T manufacturing, T-cell fitness, and therapeutic efficacy -largely through immunosenescence and altered cellular composition-it does not consistently correlate with the incidence or severity of cytokine-mediated toxicities (Ullrich et al., 2024). Similarly, sex differences may influence immune responses and the reporting of immune-related adverse events, but current evidence does not support a strong or systematic association with CRS severity across CAR-T settings (Yan et al., 2021). In contrast, clinical and mechanistic studies increasingly point to tumor burden as a dominant determinant of CAR-T- or TCE-associated adverse events, as higher target cell mass amplifies immune activation and cytokine release upon CAR-T- or TCE-engagement. Indirect support for this concept also comes from the frequent use of tumor debulking before initiation of immunotherapy, reflecting the clinical relevance of reducing tumor burden prior to treatment. Consistent with this, our recent study identified high tumor burden as a key factor associated with adverse events, whereas age and sex did not emerge as significant predictors (Scheller et al., 2025). Together, these findings suggest that, unlike infectious contexts such as COVID-19, modulating factors in CAR-T-induced CRS are more closely linked to disease- and therapy-specific parameters than to intrinsic host characteristics alone.

## 4 From irAOP to NAM-based next-generation immunotoxicity testing

While Next-Generation Risk Assessment (NGRA) has been explored in the chemical sector, its application to drugs and biotherapeutics remains underdeveloped. NGRA is exposure- and NAMs-driven, requiring knowledge of individual exposure levels to estimate potential adverse effects and to adopt a primarily *protective* rather than predictive stance. For biotherapeutics, establishing an effective and safe therapeutic exposure aligns conceptually with NGRA principles. Integrated Approaches to Testing and Assessment (IATA) provide the operational framework for NGRA by integrating diverse NAM outputs into coherent, decision-relevant evidence (OECD, 2017).

AOPs support the development of IATA, enabling systematic organization and integration of existing evidence, identification of data gaps, and iterative refinement of hazard assessments. This approach enhances regulatory decision-making by providing transparent, evidence-based support for chemical risk assessment, supporting the adoption of NAMs and reducing reliance on traditional approaches.

NAMs such as organ-on-chip systems, 3D co-cultures, and *ex vivo* slices offer human-relevant alternatives to animal testing but are fragmented. Beilmann et al. (2025) makes a strong case for integrating NAMs into drug safety assessment, moving beyond descriptive animal tests to fit-for-purpose, mechanistic, pathway-based, human-relevant risk assessment (NGRA). By categorizing drug modalities and showcasing case studies, it provides a roadmap for how NAMs can complement or even replace animal studies in preclinical development. Yet NAMs on their own remain fragmented. By embedding them into irAOP frameworks, each NAM can be mapped to specific key events, transforming isolated assays into components of IATA and NGRA. This integration provides both mechanistic anchoring and regulatory relevance: NAMs become building blocks, irAOPs the scaffolding, and IATA the operational framework that drives NGRA for drugs. Together, this offers a coherent strategy to replace descriptive animal testing with predictive, human-relevant immunotoxicology based on NAMs. In this context, irAOPs serve as natural scaffolding: they define the mechanistic map along which NAM data can be aligned and evaluated. Such structured, pathway-based integration is essential to build regulatory confidence and to translate NAMs into actionable decision-making for drug safety assessment. Accurately predicting vascular leakage requires the development of organ-specific NAMs for tissues such as the lung and gut, as endothelial responses and barrier properties differ markedly between organs.

For predicting CRS, the AOP framework enables a stepwise and mechanistically coherent mapping from activation of (resident) immune cells, through cytokine release and recruitment of additional immune cells, to downstream vascular dysfunction, hypoxia, hypotension, and organ damage. At the assay level, individual KEs can be linked to standardized NAM outputs, including cytokine concentrations, immune-cell activation markers, endothelial permeability, VE-cadherin disruption, PGE_2_ production, nitric oxide levels, or platelet-neutrophil aggregate formation. These readouts should be interpreted within the CRS-irAOP rather than as isolated endpoints, allowing complementary NAMs to be combined into an integrated assessment of pathway progression. In this way, NAM outputs can support both the quantification of individual KEs and the evaluation of response-response relationships between upstream immune activation, inflammatory amplification, endothelial dysfunction, vascular leakage, vasodilation, and other late-stage events relevant to CRS. Table 2 shows this framework by explicitly linking defined KERs to available immune-relevant NAMs, their measurable readouts, appropriate controls, regulatory readiness, and inherent limitations. In doing so, it illustrates how NAMs can be deployed in a structured, fit-for-purpose manner rather than as standalone assays.

**TABLE 2:** Framework for quantitative and semi-quantitative deployment of immune-relevant NAMs across defined CRS-related KERs**.** The table links individual KEs/KERs to measurable NAM outputs, controls or reference stressors, and current limitations relevant for translational interpretation. Inspired by Table 2 in Alb et al. (2024).

KE/KER transition	Test systems	Quantitative or semi-quantitative readouts	Controls/reference stressors	Translational relevance and limitations
KER: KE1492 “activation, immune cells” to KE1496 “increase, pro-inflammatory cytokines”	Cytokine release assay (CRA) using PBMCs, EMA accepted	IL-6, IFN-γ, TNF-α, IL-1β, GM-CSF (ELISA, multiplex); cytokine-fold change over baseline; temporal cytokine release profiles; immune activation markers (CD69, CD25 by flow cytometry)	Medium control; anti-CD3/anti-CD28; known CRS-inducing monoclonal antibodies (e.g., OKT3); non-targeting CAR-T	Limited prediction of downstream systemic effects; donor variability; insufficient modelling of feedback amplification; reduced relevance for *in vivo* CAR-T expansion dynamics
KER: Pro-inflammatory cytokines to PGE_2_ production (fever axis)	Cytokine-stimulated human monocytes/macrophages; PBMC-derived supernatant transfer assays, under qualification	PGE_2_ concentration (ELISA); COX-2 expression (qPCR/Western blot)	IL-1β/TNF-α positive control; COX-2 inhibitor (e.g., celecoxib)	No direct hypothalamic modelling; fever inferred indirectly; no standardized KE currently available in AOP-wiki
KER: Cytokines to KE 2068 “increase, endothelial dysfunction” (vascular leakage)	Human endothelial monolayers (lung, gut); endothelial-immune co-culture; vascularized organ-on-chip, under qualification	Barrier permeability (TEER decrease, dextran permeability coefficient); VE-cadherin disruption score; ICAM-1/VCAM-1 expression	TNF-α positive control; dexamethasone or anti-IL-6R	Organ specificity required; lacks systemic hemodynamic context; shear stress dependency
KER: Platelet-neutrophil interactions to KE 1916 “increase, thromboinflammation” (AOP-wiki AOP 412; KER 2458)	Human endothelial flow assays; platelet–neutrophil co-culture under flow, under qualification	Platelet-neutrophil aggregates (flow cytometry/imaging); adhesion/rolling under flow; thrombus burden/occlusion; NET markers (e.g., MPO-DNA); platelet activation (P-selectin expression); endothelial activation (ICAM-1/VCAM-1)	Basal/vehicle; TNF-α and thrombin positive controls; pathway inhibition (P-selectin/PSGL-1; integrin blockade); isotype/FMO where applicable	Donor variability; shear/platform dependence; limited systemic feedback/hemo-dynamics. NO is transient and context-dependent; quantitative thresholds are poorly defined
KER: Pro-inflammatory cytokines to PGE_2_ production (fever axis)	Cytokine-stimulated endothelial cells; macrophage-endothelial co-culture, exploratory	NO/nitrite levels; eNOS/iNOS expression, vessel diameter change or flow resistance in microphysiological systems	TNF-α/IFN-γ; NOS inhibitors	NO is transient and context-dependent; quantitative thresholds poorly define
KER: Cytokines to KE 2068 “increase, endothelial dysfunction” (vascular leakage)	Microfluidic vascular models; engineered arteriole-on-chip system, exploratory	Vessel diameter change; flow resistance; endothelial contractility	NO donors; endothelin-1	High technical complexity; limited standardization; not yet routine NAM

A critical aspect highlighted in Table 2 is the need for prototypical stressors and positive controls that anchor NAM performance to clinically relevant immune activation patterns. For CRS-related testing, such stressors may include well-characterized CRS-inducing antibodies, bispecific T-cell engagers, or CAR-T cell products, which serve as benchmarks for assay sensitivity and dynamic range. Without such reference points, interpretation of NAM outputs and comparison across platforms remains difficult.

Moreover, it is important to integrate modulating factors that contribute to the pronounced heterogeneity of CRS outcomes. Where feasible, NAMs should incorporate patient-derived cells, disease-relevant target cell burden, or stratification by sex or immune status to better reflect clinically relevant variability. While not all modulating factors can be accommodated within a single assay, their explicit consideration helps define the domain of applicability of each NAM and clarifies which aspects of CRS biology are captured or missed.

Finally, this highlights both the measurability and the current limitations of immune-relevant NAMs. Early KEs, such as immune cell activation and cytokine release, are readily quantifiable and, in some cases, already accepted or under regulatory qualification. In contrast, late KEs associated with vascular dysfunction and systemic physiological responses remain technically challenging and are largely exploratory. Explicit recognition of these strengths and gaps enables a weight-of-evidence to approach in which complementary NAMs are combined to achieve sufficient biological coverage for decision-making. Rather than serving merely as descriptive tools, such integrated NAM strategies provide a practical pathway for the iterative development, qualification, and regulatory integration of human-relevant testing approaches for CRS.

## 5 Computational challenges and opportunities in irAOP induced by biotherapeutics

**FIGURE 2:**
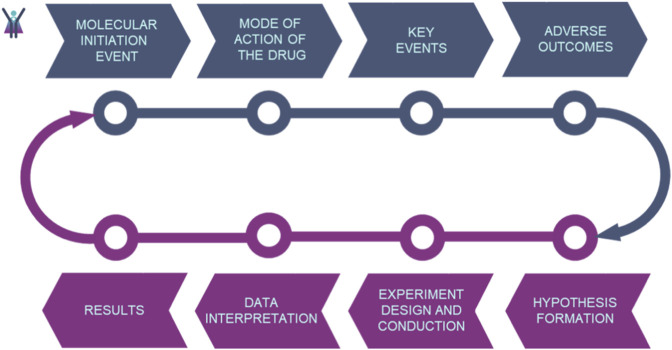
Conceptual framework for irAOP-guided prediction of immune-related toxicities. The proposed approach integrates theoretical irAOP with iterative experimental validation. Identified knowledge gaps guide hypothesis generation, experimental design, data interpretation, and refinement of the pathway, with the aim of improving the safety assessment of immunotherapies.

AOPs are an important interface between domain knowledge and computational workflows, as they can be represented in both human and machine-readable formats. This is a great opportunity for systematic integration of manually constructed and literature supported descriptions of complex mechanisms involved in adverse outcomes, and omics data generated using non-animal experimental models. From the perspective of computational and systems biology, construction of an AOP is an important step where domain knowledge is used to develop a model of a biological process (Kitano, 2002). Simulations of such a model generate hypotheses, informing wet-lab experiments, which in turn update domain knowledge and the corresponding model (Figure 2). In this context, AOP-informed QST models offer a major opportunity to transform structured mechanistic knowledge into predictive frameworks for simulating exposure-response relationships and adverse outcome trajectories. Recent concepts of lab-in-the-loop or model-in-the-loop (Qiu et al., 2025) extend this paradigm, including machine learning and artificial intelligence (AI) approaches as hypothesis generators. The key idea, however, is still a combination of expert input into a computational workflow, and its following validation in the lab.

Such coupling reinforces NAM approaches in three important aspects. First, systematically encoding knowledge in machine-readable formats requires harmonization and standardization of encoded mechanisms, mandates introduction of persistent identifiers and supports FAIRness of constructed AOPs. Second, such machine-readable content can be coupled with omics data. This in turn reveals gaps in domain knowledge, where AOP coverage does not account for important data points, and gaps in the wet lab models, where critical AOP variables have no corresponding experimental readouts. Finally, complex experimental data coupled with machine-readable AOPs and interpreted directly in the context of encoded mechanisms, which supports hypothesis validation of studied mechanisms or perturbations.

Computational implementation of AOPs is a promising approach which faces several challenges. First is the lack of state-of-the-art protocols for construction of machine-readable AOPs. Efforts related to AOP-Wiki (Ives et al., 2017) or Wiki Pathways (Martens et al., 2018) resources address this issue, however the set of proposed conventions is still insufficient for creating harmonized and interoperable computational models based on AOPs. Another challenge is maintenance of once-established AOPs. This problem is similar across the domain of computational modelling, as keeping models up to date requires substantial resources, in particular the time of domain experts to review recent literature findings and emerging datasets (Waltemath et al., 2013). Moreover, data integration into AOPs raises privacy and quality concerns. Abovementioned lab-in-the-loop concept requires a digital environment for secure and trusted processing of potentially sensitive data acquired based on patient, or patient-derived samples.

Taken together, advantages of knowledge and data integration are especially compelling in the case of irAOPs, as study of immune system modulation and its ability for self-modulation require precise model formulation, and interpretation of omics data from patients and models can be especially demanding (Mazein et al., 2025). Similarly, challenges are emphasized in the case of immune-related mechanisms, given the high speed of knowledge evolution which affects any proposed AOPs and their standards. Moreover, patient specificity of certain disorders and the need for complex readouts like trusted single cell multiomics further increase the challenge for computational workflows.

## 6 Towards regulatory acceptance of NAMs for cellular immunotherapies

EMA already recognizes that “new (*in vitro*) tools for drug development” must satisfy criteria of well-defined methodology, scientific validity (reliability and relevance), and ideally offer equal or improved predictive value compared with existing methods (Drmić et al., 2024). Although there is a draft guideline for 3Rs testing approaches, no global process for regulatory acceptance of such methods is currently fully in place, especially for safety testing of medicinal products. For complex systemic immune reactions, regulators may require a high level of evidence (e.g., reproducibility, relevance to human clinical effects, cross-laboratory validation) before accepting AOP-based risk assessments *in lieu* of conventional *in vivo* or clinical data.

Another regulatory difference is the distinction between *validation* of NAMs for chemical risk assessment and their regulatory *qualification* for defined contexts of use in the biomedical field (Hartung et al., 2024). For irAOPs to become operational, early engagement with regulators to establish “fit-for-purpose” qualification pathways will be essential. A foreseeable challenge for immune NAMs is that assay performance may be laboratory-specific, complicating qualification. However, a way to overcome that challenge would be to focus on how specific NAMs impact decision making (e.g., compound de-risking or safety margin calculation).

EMA will consider the proposed NAMs reliable when it shows high reproducibility within a laboratory (*intra-lab reproducibility*); produces consistent results across different laboratories (*inter-lab reproducibility*), demonstrates low variability when run by different operators or with minor procedural changes and is supported by a transparent and well-documented protocol. The robustness of a NAM will be judged on its reliable performance despite minor differences in materials, operators, instruments, or culture conditions. Its outputs are not overly sensitive to small, unavoidable fluctuations in the procedure, and it remains fit-for-purpose across realistic testing scenarios.

Regulatory acceptance of NAM-based approaches for predicting CRS induced by immunotherapies requires alignment with established EMA principles for method qualification and decision-making. As summarized in Box 1, a central requirement is early and continuous interaction with regulators to clearly define the fit-for-purpose context of use. This distinction is critical, as regulatory expectations differ substantially depending on whether NAMs are intended to support exploratory compound de-risking, inform dose selection, or contribute to pivotal safety decisions in early clinical development.

BOX 1 **Requirements for regulatory acceptance of a CRS-irAOP triggered by cellular immunotherapies.**

**What is needed to make a CRS-AOP triggered by cellular immunotherapies acceptable for regulatory use?**

**Fit-for-purpose context specification:** Early engagement with regulators to define the regulatory context of use (e.g., compound de-risking, dose selection, support of first-in-human decisions).

**Mechanistic evidence with (semi-)quantitative KERs:** Robust human-relevant evidence linking KEs to downstream effects, including thresholds, temporal relationships, and consideration of clinically relevant variability.

**Qualified NAMs and reference stressors:** Standardized protocols, defined endpoints, intra-/inter-laboratory reproducibility, and benchmarking against well-characterized CRS-inducing reference stressors.

**Integrated interpretation (IATA):** Weight-of-evidence integration of complementary NAMs, with transparent documentation of domains of applicability and limitations.

Beyond context specification, regulatory confidence depends on the availability of strong mechanistic evidence embedded within the irAOP framework. KEs and KERs must be supported by reproducible human-relevant data and, where feasible, expressed in quantitative or semi-quantitative terms. Defining thresholds or tipping points, for example, cytokine levels or cytokine patterns associated with endothelial dysfunction, vascular leakage, nitric oxide-mediated vasodilation, PGE_2_ production or thromboinflammatory activation, is essential to translate NAM outputs into decision-relevant information. For regulatory and clinical translation, *in vitro* KE and KER measurements need to be related to clinically relevant exposure scenarios using IVIVE, PBPK and QST modelling. Such approaches can connect administered dose, systemic exposure, target burden, immune-cell expansion and temporal cytokine dynamics to the likelihood and severity of CRS. Clinically annotated datasets and well-characterized CRS-inducing reference stressors will be essential to calibrate assay dynamic ranges, define thresholds or tipping points, and evaluate the predictive value of integrated NAM panels within a defined context of use. Importantly, the irAOP framework also provides a structured means to incorporate clinically relevant sources of variability, such as tumor burden, immune status, prior treatment history or patient-derived cellular systems, without compromising mechanistic clarity.

Finally, as highlighted in Box 1, no single NAM is expected to capture the full complexity of CRS. Accordingly, quantitative NAM outputs should not be interpreted as stand-alone predictors of CRS, but integrated across the irAOP in a weight-of-evidence framework to define domains of applicability and support specific decision contexts, such as candidate de-risking, dose selection or first-in-human risk mitigation. Regulatory applicability will therefore rely on integrated weight-of-evidence approaches in which multiple complementary NAMs are interpreted together within an IATA framework and anchored to the irAOP. Transparent articulation of what each NAM can and cannot capture, including its domain of applicability and limitations, is essential for consistent, decision-relevant use. Together, these elements provide a realistic and structured pathway for translating irAOP-informed NAM strategies into regulatory practice for cellular immunotherapies.

## 7 Conclusion & outlook

This perspective synthesizes interdisciplinary expertise from toxicology, immunology, regulatory science, and New Approach Methodology (NAM) development to address a central challenge in cellular immunotherapy: the reliable prediction of CRS in humans. We argue that current preclinical paradigms, dominated by descriptive animal models and reductionist *in vitro* assays, are insufficient to capture the complex, systemic, and patient-specific nature of excessive immune activation induced by biotherapeutics.

By proposing an irAOP leading to CRS, we provide a mechanistically anchored framework that reconciles clinical symptomatology with underlying biological processes. Central to this framework is the use of shared hub inflammatory key events, immune cell activation, recruitment, and pro-inflammatory mediator release, linked to downstream pathological consequences such as endothelial dysfunction, vascular leakage, hypotension, hypoxia, and multi-organ dysfunction. Importantly, we conceptualize CRS not as a single adverse outcome, but as a composite clinical syndrome emerging from the convergence of multiple late key events, thereby aligning mechanistic resolution with clinical reality.

Embedding human-relevant NAMs within this irAOP framework transforms isolated experimental systems into integrated, decision-relevant tools. Rather than replacing one test with another, this approach supports a weight-of-evidence strategy that enables structured interpretation of diverse datasets and transparent communication of uncertainty. Together, these concepts establish irAOPs as a unifying scaffold for predictive, human-centric immunotoxicology and lay the foundation for more rational safety assessment of cellular immunotherapies.

Looking forward, several key developments are required to translate the proposed CRS-irAOP into routine regulatory and industrial practice. First, the generation of quantitative and semi-quantitative KERs is essential. Defining thresholds, tipping points, and temporal dynamics that link early immune activation to downstream vascular and systemic effects will be critical for supporting pivotal safety decisions. This will require coordinated efforts combining advanced *in vitro* systems, physiologically based pharmacokinetic modeling, and *in vitro*-to-*in vivo* extrapolation approaches tailored to biotherapeutics.

Second, regulatory applicability will depend on early and continuous engagement with agencies to define fit-for-purpose contexts of use (Hartung, 2024). Unlike chemical risk assessment, drug development operates within a benefit-risk framework, allowing controlled degrees of immune activation. irAOPs must therefore evolve to support decision-making within this therapeutic window rather than hazard identification alone. Post-marketing pharmacovigilance data offers a unique opportunity to iteratively refine irAOPs, creating a learning cycle that continuously improves predictivity and relevance.

Third, addressing inter-individual variability remains a major frontier. Integrating modulating factors such as tumor burden, immune history, and disease-specific parameters into irAOP-informed NAMs will be crucial for capturing patient heterogeneity and advancing toward more personalized safety assessment. Advances in patient-derived models, multi-omics, and computational approaches will play a pivotal role in this endeavor.

Ultimately, the convergence of irAOPs, NAMs, and IATA provides a realistic pathway to move beyond descriptive animal testing toward predictive, mechanism-based immunotoxicology. If successfully implemented, this paradigm has the potential not only to improve CRS risk prediction, but also to accelerate innovation, enhance regulatory confidence, and better safeguard patients receiving next-generation cellular immunotherapies.

## Data Availability

The original contributions presented in the study are included in the article/supplementary material, further inquiries can be directed to the corresponding authors.
